# Self-Monitoring of Weight Loss Over Time: Secondary Data Analysis of a Randomized Controlled Trial

**DOI:** 10.2196/71604

**Published:** 2026-08-31

**Authors:** Renata Savian Colvero de Oliveira, Sharon Nabwire, Heta Merikallio, Markku J Savolainen, Janne Hukkanen, Harri Oinas-Kukkonen

**Affiliations:** 1 Software Engineering and Information Systems Unit, Oulu Advanced Research on Service and Information Systems, University of Oulu, Linnanmaa campus, Pentti Kaiteran katu 1, Oulu, 90570, Finland, 358 0442465690; 2 Medical Research Center Oulu, Research Unit of Biomedicine and Internal Medicine, University of Oulu and Oulu University Hospital, Oulu, Finland

**Keywords:** health behavior, behavior change, persuasive systems design, self-tracking, self-monitoring, weight loss

## Abstract

**Background:**

Behavior change support systems aim to shape, modify, or strengthen attitudes or behaviors without using coercion or deception. One of the main software features of persuasive system design is self-monitoring, which provides the means for users to continuously track their own performance or status, thereby facilitating goal attainment.

**Objective:**

The aim of this study is to examine whether self-input of weight (self-monitoring frequency) and its interaction with time influence weight loss in adults using a mobile health behavior change support system (mHBCSS). We hypothesized that higher self-monitoring frequency would be associated with greater weight loss, with effects varying across the intervention period.

**Methods:**

This secondary analysis used data from the intervention group of a randomized, open, waitlist-controlled trial in adults with obesity (BMI 30‐40 kg/m²). Participants used the mHBCSS for 12 months, and analyses included only participants who maintained self-monitoring for at least 6 months (N=75). Weight changes were analyzed across 9 time periods. Quantile regression (QR) was applied to examine effects on the 25th, 50th (median), and 75th percentiles of weight loss. The models included self-monitoring frequency, time periods, and their interaction. A sensitivity analysis using a multiple imputation procedure was performed to assess the robustness of the QR.

**Results:**

The time period variable was significant at the 25th weight loss percentile (QR coefficient: −1.164, 95% CI −1.453 to −0.756) and at the 50th weight loss percentile (QR coefficient: −0.603, 95% CI −0.733 to −0.493). The interaction variable was significant at the 50th (QR coefficient: −0.018, 95% CI −0.047 to −0.003) and 75th (QR coefficient: −0.036, 95% CI −0.051 to −0.027) weight loss percentiles. Self-monitoring frequency alone was not statistically significant.

**Conclusions:**

The study demonstrates that the effect of self-monitoring on weight loss is time-dependent. While a higher frequency of self-monitoring is associated with greater weight loss early in the intervention, its influence decreases as time progresses. These findings emphasize the importance of sustained engagement with self-monitoring rather than focusing solely on frequency, suggesting that interventions should incorporate strategies to maintain consistent self-monitoring use throughout the behavior change process.

## Introduction

### Self-monitoring in Digital Health

Persuasive systems have been widely used to motivate users and enhance user experiences, especially in the field of health and well-being [1]. Nevertheless, for these interventions to be practically effective, they must lead to significant modifications in behavior, resulting in better health outcomes. The concept of behavior change support systems (BCSSs) was developed to fulfill this objective. A BCSS is defined as a sociotechnical information system that aims to shape, modify, or strengthen attitudes, behaviors, or compliance without coercion or deception [2].

The implementation of BCSS is approached through the persuasive systems design (PSD) model. This model establishes the postulates (psychological and software design principles) that underlie the design of persuasive systems and emphasizes the importance of considering the context of use, the user, the technology context, and the specific features implemented in persuasive systems. Within the 4 main categories, primary task support specifically refers to design aspects meant to assist users in performing their primary objective [3].

Self-monitoring, a feature of this category, enables individuals to continuously track their own performance or status, facilitating their goal attainment [3]. It may also happen unknowingly through the system, for example, through wearable sensors [4]. To elaborate, self-monitoring is to a great extent a dynamic self-management skill that involves observing and recording one’s own behavior. It is a multicomponent procedure that involves identifying a specific behavior that needs improvement, being aware of when the behavior occurs, documenting how often and how long it happens, comparing it to a standard, and then creating a plan to change it [5]. Examples of usage include self-recognition of symptoms (such as difficulty breathing in asthma), manual recording of blood pressure, and the use of self-maintained electronic databases to monitor blood glucose levels in the management of diabetes [6].

According to the literature, the self-monitoring feature is the most widely used strategy in interventions aimed at promoting health and wellness [7-9]. For instance, a meta-analysis comprising 19 studies with a total of 2800 participants showed that interventions using self-monitoring as a behavior change strategy have the potential to decrease sedentary behavior in adults [10].

Self-monitoring is also widely used in cognitive behavioral therapy (CBT)–based interventions, where it supports the assessment, change, and maintenance of behavioral patterns. Prior qualitative evidence from mobile smoking cessation interventions indicates that CBT-based app designs are favorably perceived in terms of features, design, information quality, and engagement and are associated with changes in users’ perceptions and ways of thinking about the target behavior [11], thereby highlighting the relevance of such mechanisms beyond any single application domain. One idea within CBT’s mechanisms posits that mere cognitive awareness of one’s actions might induce modifications. Another interpretation suggests that the act of monitoring itself triggers reactivity, with individuals internally generating reinforcement or punishment. Yet another view sees monitoring as functioning like prompts or cues that influence behavior. Those explanations show that both cognitive and behavioral processes contribute to the effectiveness of self-monitoring techniques [12]. Recently, a systematic review and meta-analysis of randomized controlled trials (RCTs) evaluating mobile health (mHealth) app self-management interventions for blood pressure management found that self-monitoring was among the most commonly used behavior change techniques. These interventions led to significant reductions in both systolic and diastolic blood pressure compared to usual care, emphasizing the central role of self-monitoring across mHealth applications [13]. In the context of weight change, digital interventions have also made extensive use of self-monitoring alongside established behavior change theories. Most programs combined behavior change techniques and PSD principles, particularly self-monitoring, feedback, goal setting, and tailoring, to guide behavior [14]. Taken together, these findings highlight self-monitoring not only as a frequently applied technique but also as a core functionality embedded across digital health interventions.

Importantly, while self-monitoring is one of the most ubiquitous features in digital BCSS, it is often treated as a generic component rather than being examined as a central mechanism of change whose usage patterns and temporal dynamics may critically shape intervention effectiveness. As a result, the theoretical and empirical understanding of how self-monitoring exerts its effects in real-world digital interventions remains limited.

Another important aspect of this function is the interaction between the frequency of self-monitoring usage and time. A study that investigated how the usage patterns of mHealth app functions affect user retention found that the consistent use of the self-monitoring function helped slow the decline in app usage over time. This suggests that self-monitoring has a beneficial long-term influence on user engagement [15].

Building on this evidence, a large-scale digital weight loss study demonstrated that higher weight self-monitoring frequency was significantly associated with greater weight loss over 6 months. Clinically meaningful weight loss (≥5%) was linked to at least 3 weight SMOs per week, and analysis of outcome-based subgroups further confirmed that individuals with ≥10% weight loss engaged in weight self-monitoring more frequently than those with smaller or no weight changes. These findings reinforce the importance of self-monitoring frequency for weight management while also suggesting that different patterns of usage may correspond to different levels of success [16].

Despite substantial evidence supporting the benefits of self-monitoring for health behavior change, existing research has primarily treated self-monitoring as a static behavior change technique, offering limited insight into how its intensity and temporal dynamics function as mechanisms of change. In particular, it remains unclear whether self-monitoring frequency alone, or its interaction with time, differentially shapes outcomes such as weight loss in digitally delivered BCSSs. For example, prior research has shown that greater consistency of self-monitoring across weight, food, and exercise behaviors predicts weight loss, although adherence to self-monitoring tends to decline over time [17]. This suggests that both how often and how consistently individuals engage in self-monitoring may be critical to understanding its effectiveness.

Therefore, the objective of this study was to examine whether self-monitoring frequency and its interaction with time affect weight loss in adults using an mHBCSS. By focusing on self-monitoring as a core persuasive mechanism and modeling its temporal dynamics, this study aims to advance a more fine-grained understanding of how persuasive system features operate over time, thereby contributing to both BCSS theory and the design of more effective digital weight management interventions. We hypothesized that higher self-monitoring frequency would be associated with greater weight loss and that this association might vary over the course of the intervention, reflecting the dynamic nature of behavior change processes.

### Theoretical Background

Analyzing the persuasion context, choosing persuasive design principles, specifying software requirements, and then implementing the software are among the key processes that Oinas-Kukkonen and Harjumaa [3] suggested for creating persuasive systems for behavioral modification. By considering these processes, the aim is to place greater emphasis on improving the entire user experience and increasing user involvement over the long term [2].

The PSD model comprises 4 feature categories: primary task support, dialog support, social support, and credibility support [3]. This study focuses on primary task support (supporting weight tracking and progress monitoring) and dialog support (reminders and suggestions that encourage sustained self-monitoring). Credibility support was present in the app (verifiable expert content and links to external sources), but its analysis is beyond the scope of this paper.

The PSD framework is guided by postulates emphasizing that persuasive systems are value-laden rather than neutral, that persuasion often unfolds incrementally, that both direct and indirect routes of influence are important, and that persuasive systems should be open, unobtrusive, useful, and easy to use [3]. Grounding this study in PSD allows self-monitoring to be examined not as an isolated feature but as part of a broader persuasive system, highlighting the roles of context, temporal dynamics, and sustained engagement in supporting weight loss.

## Methods

### Sample and Mobile Health Behavior Change Support System

This study is a secondary data analysis of a randomized, open, waitlist-controlled, 2-arm trial. The trial’s design was approved by the Ethics Committee of the Northern Ostrobothnia Hospital District and the Finnish Medicines Agency, which classified the mobile health behavior change support system (mHBCSS) as an investigational medical device. The trial investigated whether an mHBCSS could help participants lose weight. It started in October 2020, and the clinical phase ended in September 2022. Participants were randomly assigned to one of two groups: (1) the intervention group (n=100), who received immediate access to the mHBCSS, and (2) the waitlist control group (n=100), who did not receive the intervention during the initial 6 months of the study period. In total, 200 adults (18‐65 years) with a BMI of 30 to 40 kg/m² were eligible to participate in the study. The primary outcome of the main trial was weight loss from baseline to the 6-month visit. Detailed information, including inclusion and exclusion criteria, was published previously [18,19]. Our study sample comprises the intervention group, with a subsample of “self-monitoring users,” that is, individuals who continued self-monitoring their weight from 6 months until the 12-month period, when access to the application was discontinued (n/N=75/100). Two participants were excluded because pregnancy and cortisone use could have influenced weight changes, introducing potential bias.

**Figure 1.**
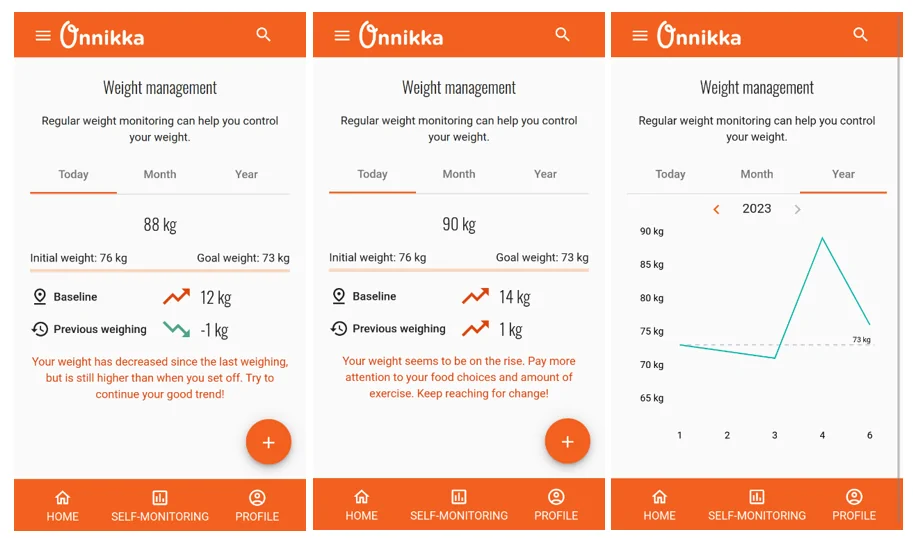
Onnikka screenshots representing the self-monitoring feature.

The app named “Onnikka,” (Onnikka Health Ltd) translated as “bus” in Finnish, provided self-monitoring features, enabling participants to monitor their own progress as shown in Figure 1. These functionalities included weight monitoring, food and exercise diaries, and a mood log where users could register their feelings and motivation during the behavior change process. After recording their weight in the application, participants also received an automated, system–generated comment about their weight loss progress (Figure 1). The gradual process of behavior change was metaphorized as a bus journey with symbolic “stops” where new content (expert-created material, videos, external links along with open-ended, multiple-choice, and slider-based interactive tasks) was delivered, aimed at guiding participants on the weight loss journey. The theoretical content was developed based on the PSD principles and the BCSS framework to support weight management among adults with obesity.

The mobile intervention applied the 3 PSD categories: primary task support (self-monitoring–related tools), dialog support (praise for progress, suggestions, and reminders for tasks), and credibility support (expertise regarding the content, verifiability by providing links to external evidence–based resources, and transparency in the privacy policy). Each Monday morning, participants received a reminder to weigh themselves. This approach aligns with the PSD principle of unobtrusiveness, providing scheduled prompts at consistent times to encourage predictability, minimize required interactions, and reduce perceived intrusiveness.

### Quantile Regression

After assessing the normality of the data using the Shapiro-Wilk test in R 4.4.0 software (R Foundation for Statistical Computing) [20], we used a nonparametric approach, quantile regression (QR) [21], to examine the influence of self-monitoring frequency on weight loss. QR allows the modeling of different quantiles of the conditional distribution of the response variable, providing a more detailed view of the effects of the explanatory variables at different points in the weight loss distribution [22]. Modeling multiple quantiles enables a more comprehensive understanding of the influence of predictors on the response distribution [23]. The rationale for using this method is that it offers greater versatility than other regression techniques in identifying associations at different points of the dependent-variable distribution [24]. Moreover, quantiles, such as the median (p=50%, where p denotes the quantile being estimated), are robust to outliers due to their emphasis on the proportion of data within a specific range, rather than on distance [25]. The QR parameter estimates the change in a specified quantile of the response variable produced by a 1-unit change in the predictor variable.

QR models were fitted to the 0.25, 0.50 (median), and 0.75 quantiles using the rq function of the quantreg package in R [26]. The model included an interaction term between self-monitoring frequency at each time period (TP) and the TPs themselves, aiming to capture how the effect of self-monitoring on weight change varied over time. Multicollinearity was detected using the variance inflation factor, with all values exceeding 5 [27] (Multimedia Appendix 1).

### Variables

#### Dependent Variable

The dependent variable was the average weight loss percentage (self-reported data). This variable represents the average percentage change in weight relative to the initial weight recorded by the user for each TP (1‐41 d, 42‐83 d, 84‐125 d, 126‐167 d, 168‐209 d, 210‐251 d, 252‐293 d, 294‐335 d, and 336‐377 d). Negative values indicate weight loss, while positive values indicate weight gain.

It was divided into 9 TPs in this analysis to balance temporal resolution and interpretability. Given that users were required to weigh themselves weekly, the intervention was organized into 6-week windows, with the exception of the first period (40 d) to ensure symmetry. This structure allowed each interval to include sufficient observations for stable QR estimates while capturing meaningful variation over time.

#### Independent Variables

The independent variables consisted of the self-monitoring frequency at each time point, the 9 TPs, and the interaction between self-monitoring and the TP (TP interaction).

#### Sensitivity Analysis

To assess the robustness of the results to missing data, a sensitivity analysis was performed using multiple imputation [28,29]. Missing data were imputed using the multiple imputation method implemented in the mice package in R, which generates multiple imputed datasets to reflect the uncertainty associated with imputation [30]. Finally, the QR was applied to each imputed dataset separately.

### Ethical Considerations

The study was approved by the Ethics Committee of the Northern Ostrobothnia Hospital District (approval: 138/2020) and the Finnish Medicines Agency, with the mHBCSS classified as an investigational medical device. The trial was registered at ClinicalTrials.gov (identifier: NCT04558801). During the conduct of the trial, the participants received oral and written information about the trial, and written informed consent was obtained.

## Results

### Sample Characteristics

In the analyzed dataset, a total of 75 participants were included, consisting of 11 males (14.7%) and 64 females (85.3%). The mean age of the participants was 48.4 (SD 9.90) years, with a median age of 49 years (IQR 41-58). Participants exhibited a mean total self-monitoring frequency of 34.6 (SD 22.4), with a median of 30 (IQR 24-40), representing the cumulative number of self-weighing inputs over the full 12-month study period. Table 1 summarizes descriptive statistics for the participants. The individuals’ detailed demographic information for the participants is shown in Multimedia Appendix 2.

**Table 1.** Descriptive statistics of self-monitoring frequency and weight by age group^a^.

Age range (y)	Frequency, n (%)	Mean self-monitoring frequency (SD)	Median self-monitoring frequency (IQR)	Mean weight change (SD), (%)	Median weight change (IQR), (%)	Mean weight at baseline (SD), (kg)	Median weight at baseline (IQR), (kg)
24‐39	14 (18.7)	26.3 (9.77)	28.5 (19.8-32.2)	−2.77 (2.77)	−3.03 (−4.95 −0.91)	97.2 (13.8)	92.6 (85.20-109.20)
40‐49	24 (32)	36.1 (28.7)	29 (24.8-40)	−3.13 (5.03)	−1.55 (−4.44 −0.14)	96.2 (12.8)	97.7 (86-102.45)
50‐59	27 (36)	39.7 (21.9)	37 (27-48)	−3.88 (3.83)	−2.72 (−5.77 −1.14)	94 (13)	91 (87.60-99.10)
60‐65	10 (13.3)	28.6 (10.7)	26 (19.2-38.8)	−2.27 (2.91)	−1.57 (−4.04 −0.39)	97.4 (13.8)	93.9 (86.10-103.10)

a Weight change is expressed as percentage change over the full 12-month study period, with negative values indicating weight loss. Self-monitoring frequency refers to the total number of self-weighing inputs over the 12-month period.

The mean weight change relative to baseline across all 9 TPs was −3.24%, which was lower than the median decrease of 2.22%, suggesting a left-skewed distribution. Moreover, the 25th percentile of weight change was −5.11%, meaning that 25% of participants lost 5.11% of their weight or more. The median weight change was −2.22%, indicating that 50% of participants lost at least 2.22% of their weight.

**Figure 2.**
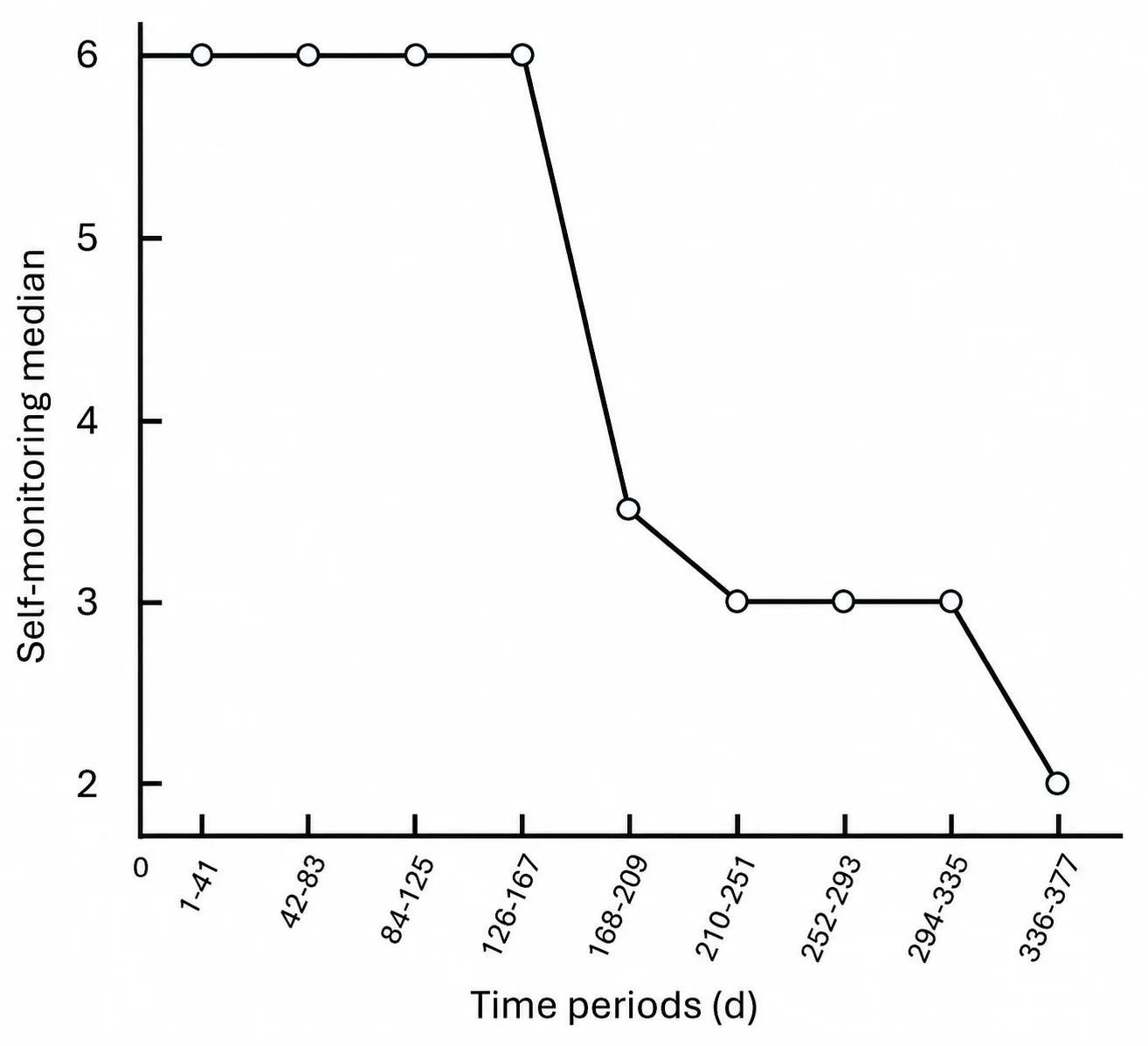
The median number of self-monitored frequency for the 9 time periods.

Figure 2 shows that during the first 4 TPs (1‐41 d, 42‐83 d, 84‐125 d, and 126‐167 d), the median self-monitoring frequency remained consistent at 6 times. In contrast, the median began to decrease in the fifth TP, reaching 3.5 times and stabilizing at 3 times during TPs 6 (210‐251 d) through 8 (294‐335 d). By TP 9 (336‐377 d), the median dropped further to 2 times.

### Percentile Analysis

In this study, the 25th, 50th, and 75th percentiles refer to participants experiencing low, median, and high percentage weight loss, respectively, across the study period.

Results of the percentile analysis in Table 2 show that for the 25th percentile (participants with relatively low percentage weight loss), the coefficient for TP was −1.164 (95% CI −1.453 to −0.756), with statistical significance. This indicates that, across TPs, participants in this group lost more weight in earlier periods, and the rate of weight loss decreased over subsequent periods.

**Table 2.** Quantile regression coefficients and 95% CIs for weight loss by percentile^a^.

Variables	Quantile regression coefficients (95% CI)		
	25th percentile	50th percentile	75th percentile
Intercept	0.53 (−1.24 to 1.64)	−0.16 (−0.78 to 0.93)	−0.10 (−0.45 to 0.42)
SMO frequency	−0.20 (−0.37 to 0.08)	0.01 (−0.17 to 0.08)	0.05 (−0.03 to 0.10)
TP^b^	−1.16 (−1.45 to −0.76)^c^	−0.60 (−0.73 to −0.49)^c^	−0.12 (−0.28 to 0.02)
TP interaction	0.02 (−0.09 to 0.05)	−0.02 (−0.05 to −0.00)^c^	−0.04 (−0.05 to −0.03)^c^

a Values are rounded to decimal places.

b TP: time period.

c Significant coefficients at the 95% CI. The 25th, 50th, and 75th percentiles refer to participants with relatively low, median, and high percentage weight loss, respectively, across all TPs.

In the 50th percentile (participants with median percentage weight loss), self-monitoring frequency alone was not significantly associated with weight change. However, both the TP variable (QR coefficient: −0.603, 95% CI −0.733to −0.493) and the interaction between self-monitoring frequency and TPs (QR coefficient: −0.018, 95% CI −0.047 to −0.003) were statistically significant. This suggests that weight loss per TP tends to be smaller in later periods at the median, and the influence of self-monitoring frequency on weight loss decreases as time progresses.

Finally, for the 75th percentile (participants with high percentage weight loss), only the interaction (QR coefficient: −0.036, 95% CI −0.051 to −0.027) was significant, suggesting that the effect of self-monitoring frequency on weight loss also diminishes over time for this group.

Overall, the influence of self-monitoring frequency on weight loss diminishes over time, with this effect being more pronounced at higher percentiles of weight loss.

The 3 intercepts (the expected initial weight loss when both the TP and self-monitoring frequency are zero) were not statistically significant.

### Multiple Imputation Procedure

To account for missing data, a multiple imputation procedure was applied, generating 5 imputed datasets. QR was performed on each imputed dataset, and the results were consistent across all imputations. Detailed results are presented in Tables 1 to 3 of Multimedia Appendix 3.

The effect of time was significant and negative, indicating that weight loss was greater in the earlier periods and declined over subsequent periods. The interaction between self-monitoring frequency and time was significant in most models, showing that the effect of self-monitoring frequency on weight loss changes over time. In contrast, self-monitoring frequency alone was not significant, highlighting that its impact can only be understood in combination with time.

## Discussion

### Principal Findings

The main finding of this study is that self-monitoring does not exert a stable effect on weight loss over time; rather, its influence is time-dependent. Specifically, the interaction between self-monitoring frequency and time was significant at the median (50th percentile) and higher (75th percentile) levels of weight loss. More frequent self-monitoring was associated with greater weight loss in the early stages of the intervention, but this effect diminished over time. Self-monitoring frequency alone did not have a significant independent effect, indicating that its impact depends on when it occurs during the behavior change process.

These results suggest that *when* and *how consistently* individuals engage in self-monitoring may matter more than how frequently they do so at a single point in time. The added value of frequent self-monitoring appears to be concentrated in the early phases of the intervention, whereas sustained engagement over time may be more relevant for maintaining weight loss, particularly among individuals achieving higher levels of success.

In contrast to prior work that has primarily examined self-monitoring as a static behavior change technique [8-10] or focused on its overall frequency [16], this study extends existing research by demonstrating that the effectiveness of self-monitoring is time-dependent and varies across different outcome levels of weight loss. By applying QR, we show that self-monitoring operates differently for individuals achieving median versus higher levels of weight loss, thereby offering a more nuanced, distribution-sensitive understanding of its role in digital weight management interventions.

The results of this study also add to the findings of Karppinen et al [31]. Based on their qualitative results, self-monitoring was perceived by users as a beneficial persuasive feature among those who achieved 5% weight loss. The interaction with time reflects and explains how self-monitoring integrates into an ongoing process of adaptation, learning, and sustained behavior change, emphasizing that its power lies in commitment rather than immediacy. This idea is supported by previous research on BCSS [32], which highlighted the importance of encouraging consistency by helping users commit to specific goals over time. Systems that reinforce user commitments are more successful in driving behavior change. That research also identified a key challenge: many individuals struggle to follow through on health-improvement goals due to a lack of self-control or willpower. Consequently, people often choose simpler solutions that fail to produce significant, long-term results because they lack strong commitments. Finally, Oduor and Oinas-Kukkonen [32] also found that primary task support, a main category of self-monitoring features, had the greatest impact on perceived competence and users’ intentions to continue, suggesting that systems encouraging goal commitment are more likely to drive lasting behavior change.

In this study, the frequency of self-monitoring alone was not significant. This is consistent with the findings of a secondary data analysis of an RCT in which individuals who used a smartphone-based system for behavioral weight loss did not experience increased weight loss despite engaging in more self-monitoring [33]. Similar findings in a prospective study showed that self-monitoring of blood glucose levels had no influence on glycemic management in individuals newly diagnosed with type 2 diabetes [34]. This evidence emphasizes our findings, indicating the importance of analyzing the interaction with time to understand the impact of self-monitoring.

Since the diminishing effect is significant at the 50th and 75th weight loss percentiles, these insights are particularly relevant for individuals in the median-to-upper ranges of weight loss. For individuals in this stage, sustained engagement with self-monitoring may demand additional support or motivational, personalized strategies. A previous study has shown that users who proactively set up a reminder have significantly higher long-term adherence to an mHealth app to improve nutrition literacy and support healthy dietary behaviors. Moreover, the relevance of the social role feature was further demonstrated by the fact that users who selected a female conversational agent had a higher likelihood of being long-term adherents [35]. A recent systematic literature review supports this evidence by showing that the most persuasive patient engagement features of mHealth apps come from dialog support (praise, rewards, reminders, suggestions, and social role) [36]. In this way, PSD features within dialog support, such as reminders, suggestions, and social role, could be effective strategies to sustain the user’s engagement. In this study, participants received 26 weekly reminders to weigh themselves during the active intervention period (the first 6 months). This structured approach ensured consistent self-monitoring during the initial phase of the study. However, after the 6-month period, these reminders ceased, potentially contributing to a decline in self-monitoring. The lack of continuous reminders may have affected participants’ adherence to routine self-weighing, leading to a decline in their capacity to maintain consistent self-monitoring behavior over time.

Moreover, self-expansion, explained as increasing positive self-content by engaging in novel and rewarding activities [37], is associated with better behavioral weight loss outcomes, including a greater likelihood of achieving a clinically significant (5%) weight loss through an internet-based behavioral weight loss intervention [38]. Self-expanding activities and/or experiences, such as learning new things about weight loss, behavioral challenges, and social interaction, might have offered alternatives to food rewards [38]. Those interpretations could lead us to infer that adding the persuasive feature “reward,” which also belongs to dialog support, could be another good strategy to be combined with self-monitoring. There is a neurophysiological basis behind this claim, since food triggers the release of dopamine in the brain, generating feelings of pleasure and reinforcement [39].

In this same direction, the reward feature was shown to be linked to the emotional state of happiness in a large-scale empirical study (n=660) aimed at investigating if and how people respond emotionally to persuasive features. Reward was the only feature that was not connected with any negative feelings (eg, anger, disgust, fear, and sadness) [40]. This is particularly relevant because people with obesity or who are overweight often turn to food as a source of reward, which can perpetuate a cycle of addiction [39]. Introducing digital rewards (eg, reward features), therefore, could represent an alternative form of stimulation, helping to replace the search for pleasure through food and facilitating the adoption of new healthy behaviors.

In practice, our study suggests that when self-monitoring is decreasing over time, it should be interpreted as a signal that the intervention needs to be strengthened or adjusted. While self-monitoring remains a valuable feature in itself, additional supportive strategies are needed to maintain or enhance its long-term effectiveness. Interventions might need to evolve to sustain engagement and impact, perhaps by introducing new elements such as social support features. Karppinen et al [31] found that the need for these features increased over the duration of an earlier web version of this same intervention. Additionally, the absence of social support was linked to lower self-monitoring, according to a qualitative study that examined the experiences and emotions related to self-monitoring in eating behaviors among participants who had completed an 18-month behavior weight reduction study [41].

Furthermore, prior research revealed that self-monitoring encourages healthy habits by inspiring reflective thinking about an individual’s behavior. As individuals tend to strive to surpass their previous main task-related records, this tendency also leads to internal competition between their previous and current attitudes [7]. However, reflection alone may not be sufficient; combining self-monitoring with other software features (such as dialog and primary task support) may help transform the need for reflection into actionable insights [42], thereby supporting long-term behavior change.

Another aspect of reducing or stopping users’ self-monitoring should be considered. Based on the results of an international survey involving users of self-monitoring technologies, Ajana [43] found that, although this feature increases motivation and encourages a healthier lifestyle, its quantitative aspects can sometimes lead to excessive behavior, which can increase pressure on the user, resulting in feelings of inadequacy, for example. Self-monitoring could also lead to health disorders (eg, in users with anorexic tendencies) and depression [7]. Other aspects on the negative side of self-monitoring are the tediousness and lack of enjoyment of the activity, reinforcing that it should be implemented alongside other strategies [7]. In the mHBCSS under investigation here, users received a brief comment about their weight loss progress after recording their weight, potentially reducing the likelihood that these last 2 factors would cause a decrease in self-monitoring. However, to overcome potential adverse effects of self-monitoring, the designers probably should adopt a comprehensive approach to promoting health and well-being, rather than monitoring just one marker of health behavior [7].

Finally, our results extend PSD theory by highlighting the temporal dimension of engagement: although self-monitoring is most impactful in the early stages, its influence diminishes over time, suggesting that persuasive systems should be designed not only to encourage initial adoption but also to sustain long-term engagement. This suggests that while PSD principles such as reminders (dialog support) can initially sustain engagement, additional strategies may be required to maintain long-term adherence. Together, these findings refine the application of PSD by emphasizing that its principles need to be considered in a temporal context. Specifically, persuasive features may be most effective in the early phases of behavior change, whereas later phases may require adaptive or complementary strategies.

### Limitations and Future Research

This study has several limitations. First, the sample size (N=75) was relatively small, which limits statistical power and may increase the risk of type II errors. Second, the majority of the sample was female (64/75, 85.3%), and the study was conducted in a single cultural and geographic context (Finland), which may limit the applicability of the results to other populations or settings.

Third, while QR allowed us to examine effects across different percentiles of weight loss, the findings are specific to the quantiles analyzed (25th, 50th, and 75th) and cannot be directly generalized to the entire population. Fourth, self-monitoring frequency was measured as the total number of self-weighing entries recorded, but this metric does not capture qualitative aspects of engagement, such as adherence to recommended behaviors, motivation, or the accuracy of self-reported data. Finally, the study design was observational, limiting causal inference between self-monitoring and weight loss outcomes.

Building on the time-dependent effects observed in this study, future longitudinal research could examine how different trajectories of self-monitoring engagement relate to distinct weight loss trajectories and how adaptive intervention strategies might sustain engagement in later phases. Given the observational design of this study, experimental or adaptive trial designs (eg, just-in-time adaptive interventions) would be valuable to establish causal relationships between self-monitoring patterns and outcomes. It might also be valuable to explore the mental and emotional impact of self-monitoring and adapt this strategy, avoiding possible negative effects and improving adherence. Additionally, the effects of age, gender, and other characteristics should be studied to personalize the interventions. Finally, future studies could examine how the framing of system-generated feedback, such as positive versus negative comments, affects motivation, consistency, and commitment to self-monitoring, providing insights to further optimize digital behavior change interventions.

### Conclusions

In this research, self-monitoring, a core functionality of the presented mHBCSS, was studied over time to understand its impact on weight loss. While self-monitoring frequency alone did not show a statistically significant independent effect, the interaction between self-monitoring frequency and time was significant at the 50th and 75th weight loss percentiles. This suggests that the potential influence of self-monitoring on weight loss may vary across the intervention period, with a stronger association observed in earlier phases that diminishes over time. These findings highlight the importance of considering the timing of behavioral engagement, suggesting that sustained self-monitoring may be more valuable for long-term effectiveness than intensity alone. Future interventions may benefit from designing strategies that encourage consistent engagement with self-monitoring tools throughout the behavior change process to maximize weight loss outcomes.

## Supplementary material

10.2196/71604 Multimedia Appendix 1 Variance inflation factor.

10.2196/71604 Multimedia Appendix 2 Demographic information.

10.2196/71604 Multimedia Appendix 3 Quantile regression analysis with multiple imputation.

## Abbreviations

BCSS: behavior change support system
CBT: cognitive behavioral therapy
mHBCSS: mobile health behavior change support system
mHealth: mobile health
PSD: persuasive systems design
QR: quantile regression
RCT: randomized controlled trial
TP: time period
