# Supplementary material for: Self-Monitoring of Weight Loss Over Time: Secondary Data Analysis of a Randomized Controlled Trial
Source: JMIR Mhealth Uhealth. 2026 Aug 31;14:e71604. doi: 10.2196/71604 (PMC13528884; doi:10.2196/71604)
Supplement: Supplementary file 1 [file mhealth-v14-e71604-s001.docx]

Appendix A. Variance Inflation Factor (VIF)

| **Variable** | **VIF (Without interaction)** | **VIF (With interaction)** |
| --- | --- | --- |
| Time periods | 1.081 | 2.317 |
| SMO frequency | 1.081 | 4.003 |
| Interaction (SMO frequency and time periods) | N/A | 4.115 |

Note: N/A – not applicable
