# Supplementary material for: Self-Monitoring of Weight Loss Over Time: Secondary Data Analysis of a Randomized Controlled Trial
Source: JMIR Mhealth Uhealth. 2026 Aug 31;14:e71604. doi: 10.2196/71604 (PMC13528884; doi:10.2196/71604)
Supplement: Supplementary file 2 [file mhealth-v14-e71604-s002.docx]

Appendix B. Demographic information

| **Characteristics** | **Frequency (n)** | **Percentage (%)** |
| --- | --- | --- |
| **Sex designated at birth** |  |  |
| Male | 11 | 14.7 |
| Female | 64 | 85.3 |
| **Marital status** |  |  |
| Unmarried | 9 | 12.0 |
| Married | 47 | 62.7 |
| Cohabitation | 11 | 14.7 |
| Divorced | 7 | 9.3 |
| Widow | 1 | 1.3 |
| **Basic education** |  |  |
| Primary school 1-6^a^ | 0 | 0 |
| Primary school 1-9^b^ | 15 | 20.0 |
| Primary School 4-9^a^ | 7 | 9.3 |
| High school graduate/ secondary school graduate | 53 | 70.7 |
| None of the earlier mentioned | 0 | 0 |
| **Professional training** |  |  |
| Professional course (at least 4 months), earlier employment course in [hidden] | 1 | 1.3 |
| Other employment course (at least 4 months) | 1 | 1.3 |
| Vocational school | 9 | 12.0 |
| University of applied sciences | 34 | 45.3 |
| University | 19 | 25.3 |
| None of the above | 11 | 14.8 |

Notes: n=75, ^a^old education system in [hidden] ^b^current education system in [hidden]
