# Supplementary material for: Self-Monitoring of Weight Loss Over Time: Secondary Data Analysis of a Randomized Controlled Trial
Source: JMIR Mhealth Uhealth. 2026 Aug 31;14:e71604. doi: 10.2196/71604 (PMC13528884; doi:10.2196/71604)
Supplement: Supplementary file 3 [file mhealth-v14-e71604-s003.docx]

Supplementary file 3

Table 1. Quantile Regression Analysis with Multiple Imputation: Models 1 to 5 for the 25^th^ percentile

|  | Model 1 | Model 2 | Model 3 | Model 4 | Model 5 |
| --- | --- | --- | --- | --- | --- |
| Intercept | -0.32  (CI: -1.62, 0.80) | 0.20  (CI: -1.26, 1.48) | -0.22  (CI: -1.26, 1.20) | 0.21  (CI: -1.32, 1.47) | 0.05  (CI: -1.31, 1.30) |
| SMO frequency | -0.09  (CI: -0.28, 0.08) | -0.16  (CI: -0.36, 0.08) | -0.09  (CI: -0.32, 0.08) | -0.18  (CI: -0.38, 0.08) | -0.14  (CI: -0.33, 0.08) |
| TP | -0.91  (CI: -1.14, -0.65)^a^ | -1.05  (CI: -1.29, -0.74)^a^ | -0.98  (CI: -1.23, -0.74)^a^ | -1.06  (CI: -1.29, -0.70)^a^ | -1.07  (CI: -1.28, -0.77)^a^ |
| TP interaction | -0.01  (CI: -0.07, 0.04) | 0.00  (CI: -0.06, 0.04) | -0.01  (CI: -0.06, 0.04) | 0.01  (CI: -0.06, 0.04) | 0.01  (CI: -0.06, -0.04) |

Notes: Rounded to decimal places. ^a^ Significant coefficients at the 95% confidence level (95% CI).

Table 2. Quantile Regression Analysis with Multiple Imputation: Models 1 to 5 for the 50^th^ percentile

|  | Model 1 | Model 2 | Model 3 | Model 4 | Model 5 |
| --- | --- | --- | --- | --- | --- |
| Intercept | -0.27  (CI: -0.81, 0.34) | -0.38  (CI: -0.93, 0.59) | -0.32  (CI: -0.83, 0.81) | -0.12  (CI: -0.78, 1.00) | -0.23  (CI: -0.81, 0.34) |
| SMO frequency | 0.01  (CI: -0.13, 0.08) | 0.02  (CI: -0.15, 0.09) | 0.02  (CI: -0.19, 0.07) | 0.00  (CI: -0.25, 0.07) | 0.01  (CI: -0.14, 0.07) |
| TP | -0.57  (CI: -0.64, -0.45)^a^ | -0.49  (CI: -0.66, -0.40)^a^ | -0.56  (CI: -0.70, -0.46)^a^ | -0.57  (CI: -0.71, -0.45)^a^ | -0.58  (CI: -0.65, -0.46)^a^ |
| TP interaction | -0.02  (CI: -0.04, -0.01)^a^ | -0.03  (CI: -0.05, -0.00)^a^ | -0.02  (CI: -0.03, -0.00)^a^ | -0.02  (CI: -0.03, 0.00) | -0.02  (CI: -0.03, -0.01)^a^ |

Notes: Rounded to decimal places. ^a^ Significant coefficients at the 95% confidence level (95% CI).

Table 3. Quantile Regression Analysis with Multiple Imputation: Models 1 to 5 for the 75^th^ percentile

|  | Model 1 | Model 2 | Model 3 | Model 4 | Model 5 |
| --- | --- | --- | --- | --- | --- |
| Intercept | 0.16  (CI: 0.00, 0.58)^a^ | 0.01  (CI: -0.15, 0.43) | 0.07  (CI: -0.13, 0.44) | 0.00  (CI: -0.17, 0.45) | 0.11  (CI: -0.10, 0.51) |
| SMO frequency | 0.03  (CI: -0.05, 0.04) | 0.04  (CI: -0.04, 0.04) | 0.04  (CI: -0.04, 0.04) | 0.04  (CI: -0.04, 0.04) | 0.03  (CI: -0.05, 0.07) |
| TP | -0.23  (CI: -0.30, -0.19)^a^ | -0.16  (CI: -0.26, -0.10)^a^ | -0.19  (CI: -0.27, -0.16)^a^ | -0.16  (CI: -0.26, -0.11)^a^ | -0.20  (CI: -0.30, -0.13)^a^ |
| TP interaction | -0.03  (CI: -0.03, -0.01)^a^ | -0.03  (CI: -0.05, -0.01)^a^ | -0.03  (CI: -0.04, -0.01)^a^ | -0.03  (CI: -0.05, -0.01)^a^ | -0.01  (CI: -0.03, -0.00)^a^ |

Notes: Rounded to decimal places. ^a^ Significant coefficients at the 95% confidence level (95% CI).
